# Biological Activity of Lenalidomide and Its Underlying Therapeutic Effects in Multiple Myeloma

**DOI:** 10.1155/2012/842945

**Published:** 2012-08-02

**Authors:** Roberta Martiniani, Valentina Di Loreto, Chiara Di Sano, Alessandra Lombardo, Anna Marina Liberati

**Affiliations:** Department of Oncohematology, University of Perugia, Santa Maria Hospital, 05100 Terni, Italy

## Abstract

Lenalidomide is a synthetic compound derived by modifying the chemical structure of thalidomide. It belongs to the second generation of immunomodulatory drugs (IMiDs) and possesses pleiotropic properties. Even if lenalidomide has been shown to be active in the treatment of several hematologic malignancies, this review article is mostly focalized on its mode of action in multiple myeloma. The present paper is about the direct and indirect antitumor effects of lenalidomide on malignant plasmacells, bone marrow microenvironment, bone resorption and host's immune response. The molecular mechanisms and targets of lenalidomide remain largely unknown, but recent evidence shows cereblon (CRBN) as a possible mediator of its therapeutical effects.

## 1. Introduction

**Figure 1:**
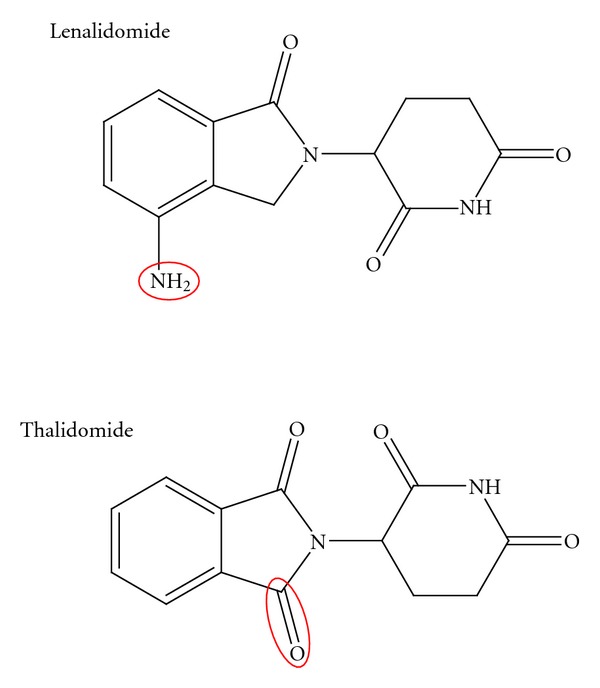
Lenalidomide and thalidomide structure.

Lenalidomide and pomalidomide are synthetic compounds derived by modifying the chemical structure of thalidomide [1]. In particular, as shown in Figure 1, lenalidomide has been synthesized from the structural bone of thalidomide molecule. Lenalidomide has been developed by adding an amino group (NH2–) at 4th position of phthaloyl ring and by removing the carbonyl group (C=O) of the 4-amino-substituted phthaloyl ring. This drug is the result of the pressing need to develop molecules with enhanced immunomodulatory and antitumor activity in comparison to thalidomide. Lenalidomide, which possesses pleiotropic properties, belongs to the second generation of immunomodulatory drugs (IMiDs).

Lenalidomide and its parental molecule thalidomide have shown therapeutical activity in various malignancies [2–21].

The US Food and Drug Administration (FDA) first approved lenalidomide for the treatment of patients suffering from 5q-myelodysplastic syndrome [22]. However, because of the proven activity of thalidomide in multiple myeloma (MM), the clinical activity of lenalidomide has been evaluated more extensively in this neoplasia [7–12], in respect to other B-cell neoplasia. The favourable toxic profile of lenalidomide and its antitumor activity emerged from phase I and phase II studies in relapsed or refractory MM patients [23–25]. These encouraging results led to the design of two large, phase III, multinational, randomized, double-blind, placebo-controlled, registration trials (MM-009 in US and Canada and MM-010 in Europe, Australia, and Israel) in this setting of patients. In both studies, patients were randomly assigned to receive 25 mg of lenalidomide or placebo on days 1 to 21 of 28-day cycles plus dexamethasone (40 mg on days 1 to 4, 9 to 12 and 17 to 20 for the first four cycles, then only on days 1 to 4). The results of these trials have shown the superiority of lenalidomide-dexamethasone combination compared to placebo-dexamethasone in terms of time to progression (11,1–11,3 months versus 4,7 months in the lenalidomide and in the placebo group, resp., *P* < 0, 001), overall survival (in MM-009: 29,6 months versus 20,2 months in the lenalidomide and in the placebo group, resp., *P* < 0, 001, in MM-010: hazard ratio for death 0,66, *P* = 0, 03) and overall response rate (60,2–61% versus 19,9–24% in the lenalidomide and in the placebo group, resp., *P* < 0, 001). At a median followup of 48 months for surviving patients, a pooled update analysis of these studies has shown a significant benefit in overall survival (38 versus 31,6 months, *P* < 0, 045) for those patients initially randomized to be treated with lenalidomide-dexamethasone combination [8, 26]. It should be emphasized that the improved survival associated to lenalidomide-dexamethasone treatment was retained despite 47,6% of patients, who were initially randomized to placebo dexamethasone, received lenalidomide-based therapies after disease progression or study unblinding [27]. More recently, several studies have compared the activity of lenalidomide combined with high or reduced dose of dexamethasone in newly diagnosed MM patients [28, 29]. The results of these experiences are in favour of low dose of dexamethasone. Furthermore, clinical experience with lenalidomide indicates that early use in MM therapy is associated with a higher response rate and, possibly, prolonged survival [30]. To further improve the outcome of lenalidomide, combination regimens (BiRd, VRD, RAD, VDCR, and VRDD) [31–35] have been evaluated or are under investigation in both old and young MM patients, in transplant and non transplant settings. 

MM has been chosen for this article with the purpose of showing our current knowledge on the mechanisms of antitumor activity of lenalidomide. Some of these actions are operative in other diseases too.

## 2. Biological Features of Multiple Myeloma

To understand the therapeutic activity of lenalidomide in MM, the knowledge of the pathophysiology of this disease and the complex crosstalk between malignant plasma cells (PCs) and their microenvironment in tumor growth and progression is relevant. In addition, the notion that survival of neoplastic cells is dependent on the escape from the host's antitumor immune response can help to explain the therapeutic role of lenalidomide.

Two major pathways are involved in the early pathogenesis of MM [36, 37]. Nearly half of these tumors are nonhyperdiploid and mostly are characterized by immunoglobulin H (IgH) translocations that involve five recurrent chromosomal loci, including 11p13, 6p21, 4p15, 16p23, and 20p11, which result in the dysregulated expression of an oncogene [36, 38]. These genetic lesions are responsible, at least in part, for anen hanced proliferative capacity of malignant PCs. In fact, the translocations lead directly (11q13 cyclin D1 and 6p21 cyclin D3) or indirectly (4p16, 16p23, 20p11 cyclin D2) to cyclin D dysregulation. In hyperdiploid tumors, cyclin D1 or less often cyclin D2 is usually dysregulated too [38]. Cyclin D, together with CDK4 and CDK6, regulates G1-S cell cycle progression by phosphorylating and inactivating retinoblastoma protein (RB). This reaction is inhibited by the CDK inhibitors p161INK4a and p181INK4c. These molecules can undergo mutations in MM and, in addition to cyclin D [39–41] dysregulation, can further facilitate the proliferation of the neoplastic clone.

Malignant PCs reside in the BM microenvironment which comprises physical and soluble factors. Physical elements of BM include extracellular matrix (ECM), glycoproteins, hemopoietic stem, progenitor, and precursor cells, as well as B, T, and NK lymphocytes, bone marrow endothelial cells, osteoclasts, and osteoblasts and bone marrow stromal cells (BMSCs). Tumor cells adhere to ECM proteins and BMSCs. These interactions are responsible for tumor cell localization in the BM milieu and moreover for multiple biologically relevant sequelae [37, 42]. Adhesion molecules, including CD44, very late antigen 4 (VLA-4), very late antigen 5 (VLA-5), leukocyte function-associated antigen-1 (LFA-1, CD11a), neural cell adhesion molecule (NCAM, CD56), intercellular adhesion molecule-1 (ICAM-1, CD54), syndecan (CD138), and monocyte chemoattractant protein-1 (MPC-1), mediate adhesion of malignant PCs to either ECM proteins or BMSCs [37, 42] Table 1.

**Figure 2:**
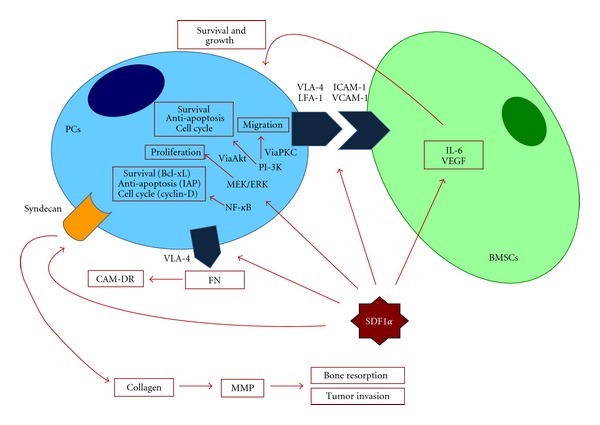
SDF-1*α* actions and its functional sequelae.

The initial homing of neoplastic cells to the BM milieu is mediated by the binding of the stromal-derived growth factor (SDF-1*α*), present in the BM, to its receptor CXCR4, expressed by malignant PCs. High serum concentrations of SDF-1*α* correlate with a more aggressive disease. This event is the consequence of the effect of this chemokine on IL-6 and VEGF production by BMSCs. These cytokines promote PC growth and survival [43]. Furthermore, SDF-1*α* modulates the expression of adhesion molecules on PCs (VLA4 and LFA-1) and BMSCs (VCAM-1 and ICAM-1) and favours the adherence between these cells. Syndecan and VLA-4, expressed on malignant PCs, mediate their adhesion to collagen and fibronectin, respectively [44, 45]. Finally, adhesion of malignant PCs via syndecan to collagen induces matrix metalloproteinase-1, thereby promoting bone resorption and tumor invasion, while binding via VLA-4 to fibronectin is responsible for cell adhesion-mediated drug resistance (CAM-DR) [44, 45] (Figure 2).

**Figure 3:**
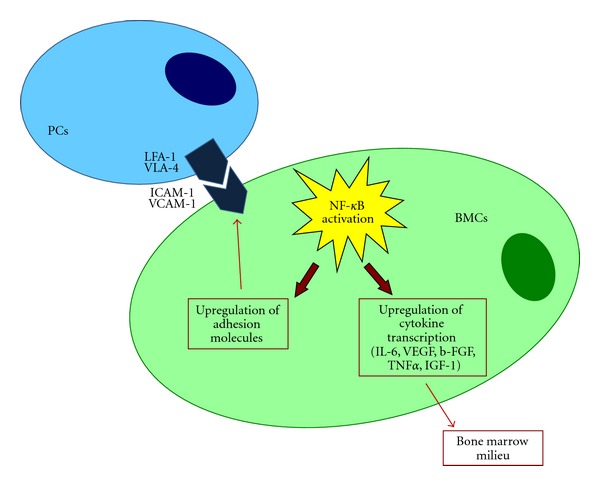
NF-kB activation and its functional biological sequelae.

Moreover, adhesion of PCs to BMSCs triggers, in these latter cells, the nuclear factor kappa-light-chain-enhancer of activated B cells (NF-kB) which results in both further upregulation of adhesion molecules, transcription and secretion of interleukine-6 (IL-6) [46] and other cytokines (vascular endothelial growth factor (VEGF), basic fibroblast growth factor (b-FGF), tumor necrosis factor-*α* [TNF*α*] and insulin-like growth factor-1 [IGF-1]) within the BM milieu [47, 48]. (Figure 3).

**Figure 4:**
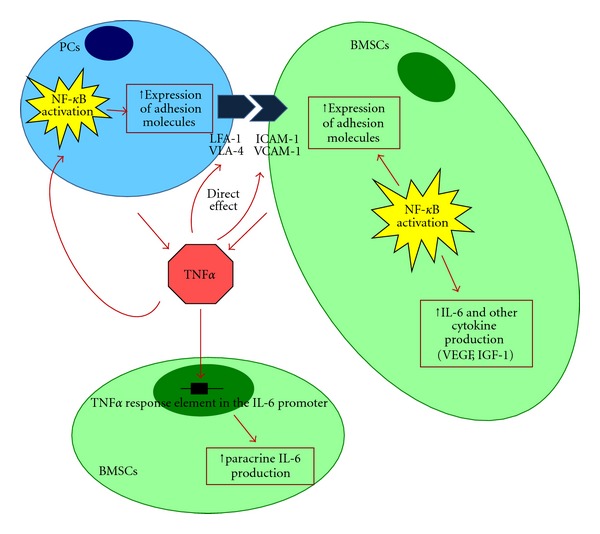
Induction of IL-6 secretion by TNF*α* and NF-kB activation.

In detail, IL-6 is a critical growth factor for normal B-cell and PC development. IL-6 is primarily produced by BMSCs and by only a few malignant PCs [49]. TNF*α* is secreted by both malignant PCs and BMSCs. It does not induce growth and survival of the neoplastic clone directly, but it binds to a TNF*α* response element of the IL-6 promoter in BMSCs inducing paracrine production of IL-6 [49, 50]. Furthermore, TNF*α* secreted by malignant PCs activates NF-kB pathway, which results in additional upregulation of adhesion molecules (CD49d, an integrin alpha subunit and ICAM-1) on both tumor PCs and BMSCs [50]. The final effects of this loop consist in additional paracrine secretion of IL-6, as well as that of IGF-1 and VEFG by BMSCs and in induction of CAM-DR [44, 46] (Figure 4).

Cytokine secretion in BMSCs is also upregulated by PC-derived transforming growth factor *β* (TGF-*β*) and VEGF [48]. This event, in turn, induces BMSCs to produce further TNF*α*, VEGF and b-FGF. Overall, these events lead to the generation of a vicious circuit responsible for continuously increased cytokine production and malignant PC clone expansion.

**Figure 5:**
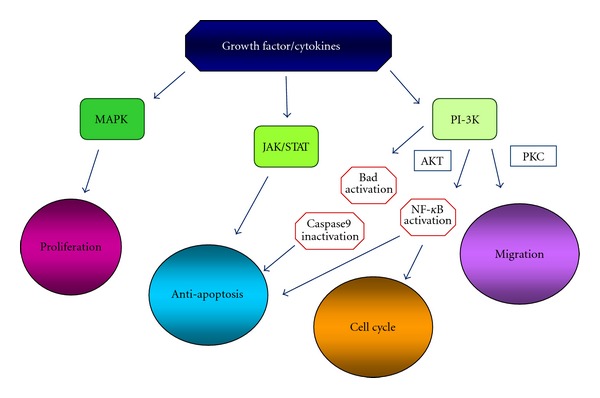
Signaling pathways activated by BM cytokines.

Binding of the cytokines to their receptors, expressed on malignant PCs, leads to activation of mitogenic/antiapoptotic pathways (mitogen actived protein kinase [MAPK], janus kinase/signal transducer and activator of transcription [JAK/STAT], phosphatidylinositol 3-kinases/protein kinase B [PI-3K/Akt] and inhibitor of nuclear factor kappa-B kinase [IKK/NF-kB]) [37], which promote cell proliferation, survival, cycle progression and migration. Survival is also mediated by increased transcription of antiapoptotic molecules (B cell lymphoma gene-2 [Bcl-2] family members such as B-cell lymphoma-extra large [Bcl-xL], myeloid cell factor-1 [Mcl-1] and caspase inhibitor such as Fas-Associated protein with Death Domain-like [FADD-like] IL-1*β*-converting enzyme (FLICE) inhibitor protein (FLIP) and cellular inhibitor of apoptosis protein 2 (cIAP-2)) in malignant PCs which act along with disregulated cyclins whose expression is further upregulated by NF-kB activation [51–54]. Overall, cytokines present in the BM milieu, reflecting the PC-BMSC bidirectional interactions, mediate growth (IL-6, IGF-1, VEGF), survival (IL-6, IGF-1), drug resistance (IL-6, IGF-1, VEGF), and migration (IGF-1, VEGF, SDF1*α*) of neoplastic cells as well as angiogenesis (VEGF, b-FGF) (Figure 5).

Because of its pleiotropic properties, lenalidomide interferes with several pathogenetic relevant moments associated to different clinical phases of MM. 

First of all, lenalidomide upregulates the cyclin dependent kinase inhibitor 1 (p21/waf1), a key cell cycle regulator that modulates the activity of cyclin dependent kinase (CDKs) [55]. Recently it has been demonstrated that lenalidomide mediates the increased expression of p21 by an epigenetic mechanism [56]. Lenalidomide reduces histone methylation and increases histone acetylation of the p21 promoter, thus enhancing transcription factor access to the DNA. In addition to upregulation of p21, lenalidomide-mediated growth inhibition has been demonstrated to be associated with the induction of CDK inhibitors p15, p16 and p27 and the early response transcription factors Egr1, Egr2 and Egr3 [57]. In MM derived cell lines, U266 and LP-1, reduction in CDK2 activity has been demonstrated after exposure to lenalidomide [55].

Lenalidomide inhibits the production of proinflammatory cytokines TNF-*α*, IL-1, IL-6, and IL-12 and increases the secretion of anti-inflammatory cytokine IL-10. IMiDs have an opposite effect on IL-12 production, depending on the different type of stimulation on peripheral blood mononuclear cells [58].

Lenalidomide also downregulates adhesion molecules. This effect is mediated by the inhibition of TNF*α* production [50]. Thus, lenalidomide ultimately suppresses a positive feedback loop which upregulates the expression of cell surface adhesion molecules on both BMSCs and malignant PCs. Moreover the downregulation of PC adherence to BMSCs reduces the production of cytokines by these cells (IL-6, VEGF, IGF-1) which, as previously indicated, are responsible for thegrowth and survival of neoplastic clone. Lenalidomide also reduces the production of IL-6 by a direct action [59].

Increased micro-vascular density has been reported to correlate with MM-progression [60]. VEGF is produced by malignant PCs and BMSCs and accounts, at least in part, for increased angiogenesis in the BM of MM patients [60]. All IMiDs, including lenalidomide, possess antiangiogenic activity. This effect appears to occur via the modulation of TNF*α*, VEGF and b-FGF, which regulate endothelial cell migration, rather than cell proliferation. Antiangiogenesis by lenalidomide correlates with reduced Akt phosphorylation in response to both VEGF and bFGF [61]. Beyond the anti-angiogenesis, the lenalidomide induced-inhibition of VEFG and bFGF production determines other biological effects. In fact, these growth factors upregulate the production by BMSCs of pro-inflammatory cytokines including IL-6.

Apoptosis is triggered by the activation of both extrinsic and intrinsic pathways. Besides, the success of this process is also related to the down-regulation of inhibitor of apoptosis protein (IAP) activity. In malignant PCs, caspase 8 is activated in response to extracellular apoptosis-inducing ligand (i.e., FADD) [62]. Lenalidomide is able to induce caspase 8 activity which in turn results in increased malignant PC apoptosis [62]. Bcl-2 homology domains (BH3) interacting domain death agonist (Bid) can mediate a cross-talk of apoptotic signaling from caspase 8 to caspase 9 [63]. On the other hand,dexamethasone induced apoptosis in MM cells is associated with caspase 9 activation and release of second mitochondrial-derived activator of caspases (Smac) [64]. Moreover, long term treatment of malignant PCs with lenalidomide determines a downregulation of NF-kB activity, which results in a reduction of antiapoptotic proteins including cIAP2 [65] and FLIP [66]. Thus, lenalidomide induced apoptosis is the result of multiple effects consisting in the direct upregulation of caspase 8 activity, indirect upregulation of caspase 9 and the downregulation of NF-kB activity which, in turn, determines the inhibition of FLIP and cIAP2 and antagonizes prosurvival effects mediated by several cytokines (IL-6 and IGF-1). NF-kB is activated by IL-6 and determines the production of antiapoptotic proteins. Consequently, lenalidomide, by inactivating NF-kB, inihibits the antiapoptotic activity induced by IL-6.

Defective host immune surveillance has a central role in the survival of malignant PCs [67]. The mechanism responsible for myeloma cell tolerance includes the immunosuppressive activity of cytokines such as TGF-*β* derived by malignant PCs [68], reduced numbers of CD4+ T-cells [69], impaired cytotoxic CD8+ T-cell responses [70], defective antigen presentation, disfunction of human natural killer-T (NK-T) and natural killer (NK) [71, 72] cells as well as resistance to NK cell lysis [73].

Lenalidomide acts at different levels in the immune system by modifying cytokine production, improving T-cell activity, regulating T-cell co-stimulation and augmenting the NK-T- and NK-cell cytotoxicity. Lenalidomide enhances the cytolytic activity of antigen-specific CD8+ T-cells. This effect has been demonstrated in a dendritic cell/CD8+ T-cell in vitro co-culture system. It appears to be mediated by IL-2 induced expansion of antigen-specific memory effector CD8+ T-cells [74].

T-cell activation requires the presentation of the peptide fragments by antigen presenting cell (APC) to the T-cell receptor (TCR). Moreover to generate an effective response against the antigen, a secondary interaction is required [75]. This is mediated by the B7 family molecules on APC and CD28 molecule on the T-cell surface and provide the costimulatory signal that augments and potentiates T-cell proliferation, differentiation and survival followed by IL-2 and IFN*γ* production. In MM patients the number of dendritic cells (DCs) is normal, but CD80 (B7-1) expression may fail to be upregulated in the presence of trimeric human CD40-ligand (HU-CD40LT) because of the negative effect of tumor-derived TGF-*β* or IL-10 [76]. Impairment of T all activation by DCs is also mediated by IL-6 [77] and VEGF [78] of PC or BMSC origin. IMiDs including lenalidomide are only able to stimulate T-cells that have been partially activated by either anti-CD3 or DCs [75]. Lenalidomide induces the proliferation of partially activated CD3+ T cells obtained from human PBMC. T-cell proliferation is associated with increased IL-2 and INF*γ* production. The mechanism of   T-cell co-stimulation by lenalidomide involves increased transcriptional activity of activated protein-1 (AP-1), a driver of IL-2 production [79]. In addition, this drug abrogates the requirement of a secondary co-stimulation signal from APCs to allow T-cell activation. In fact, it acts on T-cells via the B7-CD28 costimulatory pathway directly inducing tyrosine phosphorylation of CD28 on T-cells leading to the activation of downstream targets such as PI3K-signaling pathway and the nuclear translocation of the nuclear factor of activated T cells-2 (NFAT-2) [75, 80].

NK-T- and NK-cells belong to distinct lymphocyte lineages. However, these cells share striking similarities such as the expression of the same set of receptors (NKR-P1 and Ly49) and the capacity to rapidly release, without prior sensitization, INF*γ* and IL-4 (NK-T) or INF*γ* alone (NK) [81, 82]. IL-12 can modulate both NK-T-cells [83, 84] and NK-cells [85] to release INF*γ* and exert natural cytotoxicity. NK-T-cells are distinct lymphocytes, which often use a restricted T cell receptor (V*α*24-V*β*11) that recognizes glicolipid ligands in the context of the major histocompatibility class 1-like CD1d molecule. The anti-tumor properties of these cells include a direct cytotoxic effect of neoplastic cells, INF*γ* production and interaction with DC expressing glicolipid ligands. Lenalidomide increases the NK-T-cell expansion mediated by DCs loaded with *α* GalCer and INF*γ* production from NK-T-cells [86]. Because of the cross-talk between NK-T- and NK-cells, NK-T-cells transact with NK-cells. This network of activation later involves B and T cells indicating the sequential recruitment of distinct and adaptive effector lymphocytes [87]. Lenalidomide might potentiate the function of these other immune cells, using the transactivation mediated by NK-T-cells.

Lenalidomide not only increases NK-cell proliferation, but also potentiates natural and antibody dependent cellular cytotoxicity (ADCC) of NK-cells [88]. These effects are mediated by lenalidomide-induced IL-2 production by T cells. More in detail, lenalidomide triggers PI3K activation of AP-1 and related increased IL-2 secretion by T cells [88]. IL-2 in turn activates NK-cells.

 Bone remodelling is a tightly regulated process. The binding of receptor activator of NF-kB ligand (RANKL), on BMSCs and OBLs, to its receptor RANK, on mature OCLs and their precursors, stimulates OCL late differentiation and activity. Osteoprotegerin (OPG), a decoy receptor for RANKL, is produced by OBLs. OPG inhibits RANK-RANKL interaction, thus suppressing osteoclastogenesis [89]. Several cytokines and chemokines [IL-6, IL-1*α*, IL-1*β*, IL-11, macrophage-colony stimulating factor (M-CSF), TNF-*α*, TNF-*β*, macrophage inflammatory proteins-1*α* and -*β* (MIP-1*α*, -*β*) and VEGF], which possess pro-osteoclastogenic activity, as previously mentioned, are present in the BM milieu. Other molecules, as SDF-1*α*, IL-3 and hepatocyte growth factor (HGF), secreted by both malignant PCs and BMSCs, stimulate the expression of RANKL by BMSCs and thus enhance osteoclastogenesis. In MM, OPG production is downregulated. In addition, malignant PCs internalize and degradate OPG. This vicious cycle determines an increased RANK-RANL binding, augments OCL differentiation and proliferation and favours bone resorption [90]. Moreover, OBL activity is impaired in MM. In fact, malignant PCs suppress OBL differentiation and induce mature OBL apoptosis through the production of dickkopf-1 (DKK-1) and soluble frizzle-related protein-2 (sFRP-2). These molecules inhibit the Wingless-type (Wnt) signaling pathway, which promotes OBL differentiation. Other molecules, such as IL-7, IL-3 and TGF-*β*, overexpressed in MM BM milieu, also downregulate the OBL maturation [91].

Lenalidomide has been reported to reduce osteoclastogenesis in MM [91]. This effect is achieved in a dose-dependent manner through the inhibition of the transcription factor PU.1 and extracellular signal-regulated kinase (ERK). The first one is an early activator of osteoclastogenesis; the second one plays a key role in OCL survival and differentiation. In MM patients, after treatment with lenalidomide, OPG levels were significantly higher than baseline (*P* < 0, 05), whereas RANKL production was inhibited, so lenalidomide has been confirmed to reduce the serum markers of bone lytic disease.

Although all the above mentioned mechanisms explain the direct and indirect anti-myeloma effect of lenalidomide, the precise molecular mechanisms and targets through which this molecule exerts its effects remain not completely understood.

A seminal paper has recently identified cereblon (CRBN) as a primary target of thalidomide teratogenecity [92] and moreover an essential element for response to lenalidomide [93]. Human CRBN is a 51 kDa protein that is localized in cytoplasm, nucleus and peripheral membrane of cells in testis, spleen, prostate, liver, pancreas, placenta, kidney, lung, skeletal muscle, ovary, small intestine, peripheral blood leukocytes, colon, brain and retina [94]. CRBN links to DNA damage-binding protein 1 (DDB1) [92]. DDB1 is a nucleotide excision repair protein which binds to DDB2 leading to set up a heterodimer. It is part of the cullin-4 (Cul4)-based E3 ubiquitin protein ligase complex. This complex is formed by DDB1, Cul4 (Cul4A and Cul4B), regulator of cullins-1 (Roc1) and a substrate receptor. Cul4-based E3 ubiquitin protein ligase complex plays a relevant role in cell cycle regulation, carcinogenesis and embryogenesis [95, 96]. CRBN is a part of the Cul4-based complex and it competes with DDB2 in binding to DDB1. CRBN-complex has auto-ubiquitination properties, which are inhibited by thalidomide, as shown in in vitro-studies.

Several in vitro studies have shown that CRBN is also the target molecule of lenalidomide activity. 

Zhu et al. have clearly demonstrated in human MM cell lines (HMMCLs) the central role of CRBN in sensitivity and resistance to lenalidomide and have identified interferon regulatory factor-4 (IRF-4) as one of the downstream targets of CRBN. IRF-4 has previously reported to also be a target of and downregulated by lenalidomide [93].

Lopez-Girona et al. have demonstrated that lenalidomide binds to CRBN-DDB1 complex in a dose-dependent manner and with a ten-fold higher affinity than thalidomide. Moreover, after reducing CRBN expression by short interfering RNAs (siRNAs) in activated human T cells, lenalidomide has increased IL-2 and TNF-*α* production by these cells, thus suggesting that some immunomodulatory effects of lenalidomide are mediated by CRBN complex. This study has also shown that induction of p21/waf1 cyclin-dependent kinase inhibitor protein is prevented in absence of CRBN expression, indicating a role of CRBN in mediating antiproliferative effects of lenalidomide [97].

Heintel et al. have found a significant relationship between CRBN expression and response to lenalidomide in 44 MM patients. In fact, CRBN expression resulted three times higher in responding patients compared to non-responders. Moreover, this study has shown a clear correlation between CRBN levels and quality of response. CRBN expression was lower in patients with stable or progressive disease and higher in patients with complete remission or partial responses [98]. 

The data emerging from in vitro studies as well as the in vivo findings about the role of CRBN in lenalidomide action wait to be confirmed. 

## 3. Conclusions

IMiDs including lenalidomide have proven therapeutically effective molecules in several malignant diseases characterized by different hystogenetic origin of neoplastic cells, as well as by distinct phatogenetic pathways. Notwithstanding these differences IMiDs activity in the diverse neoplasia can be traced back to the pleiotropic mechanism of these molecules.

Lenalidomide exerts a direct antitumor effect, interferes with the tumor microenvironment and enhances the host's antitumor immune responses. In MM, because of the complex bidirectional cross-talk between malignant PCs and the BM milieu including the BMSCs, the ECM proteins and the multitude of cytokines secreted in the BM milieu, the final effects of lenalidomide are the results of additional or synergic actions on different relevant pathogenetic events operating in this disease.

In addition, lenalidomide activates caspase 8 and downregulates NF-kB activity induced by cytokines secreted in the BM milieu. This in turn determines reduced expression of antiapoptotic proteins. Thus, relevant in the lenalidomide apoptosis is also the modulation induced by this drug on adhesion molecules on PCs and BMSCs as well as on cytokines production. The anti-angiogenetic well known properties of IMiDs, including lenalidomide, might be relevant in MM as increased microvascular density has been reported to be associated with disease progression. Furthermore, lenalidomide acts on different host's effector immune cells. However the immune-mediated antitumor activity well defined in vitro are not completely correlated with the clinical outcome because of the complex immunosuppressive activity of underlying disease as well as of conventional antitumor drugs.

Finally, lenalidomide downregulates bone resorption. 

The molecular mechanisms and targets of lenalidomide remain largely unknown. However, CRBN has recently been identified as the possible central mediator of lenalidomide activity and IRF-4 as a downstream molecule of CRBN action. Lenalidomide resistance in MM cells which, despite CRBN depletion, are able to restore their IRF-4 levels, suggest the existence of alternative pathways.

## Figures and Tables

**Table 1:** “Crosstalk” between PC and BMSC.

*Adhesion molecules*
Very late activation antigens-4 (VLA-4)
Lymphocyte function-associated antigen-1 (LFA-1)
Vascular cell adhesion molecule-1 (VCAM-1)
Intercellular adhesion molocule-1 (ICAM-1)
Syndecan-1

*Cytokines*
Tumor necrosis factor-*α* (TNF-*α*)
Transforming growth factor-*β* (TGF-*β*)
Vascular endothelial growth factor (VEGF)
Fibroblast growth factor-2 (FGF-2)
Hepatocyte growth factor (HGF)
Angiopoietin-1 (Ang-1)
Interleukin-6 (IL-6)
Insulin-like growth factor (IGF-1)

*Proteasi*
Matrix metalloproteinases -2 and -9 (MMP-2 e MMP-9)

*Chemokine*
Macrophage inflammatory protein-1 (MIP-1)
Stromal derived factor-1 (SDF-1)
